# Structural and Optical Properties of New 2-Phenylamino-5-nitro-4-methylopyridine and 2-Phenylamino-5-nitro-6-methylpyridine Isomers

**DOI:** 10.3390/ijms262311522

**Published:** 2025-11-27

**Authors:** Patrycja Godlewska, Jerzy Hanuza, Jan Janczak, Radosław Lisiecki, Paulina Ropuszyńska-Robak, Lucyna Dymińska, Wojciech Sąsiadek

**Affiliations:** 1 Department of Bioorganic Chemistry, Faculty of Production Engineering, Wroclaw University of Economics and Business, 118-120 Komandorska Str., 53-345 Wrocław, Poland; 2 Institute of Low Temperature and Structure Research, 2 Okólna Str., 50-422 Wrocław, Poland

**Keywords:** 2-*N*-phenylamino-(4 and 6)-methyl-5-nitropyridines, XRD crystal structure, IR and Raman studies, electron absorption and luminescence spectra, DFT calculations

## Abstract

Two new 2-*N*-phenylamino-5-nitropyridine—4-methyl (2PA5N4MP) and 2-*N*-phenylamino-5-nitropyridine-6-methyl (2PA5N6MP) isomers were synthesized and comprehensively characterized by single-crystal X-ray diffraction, IR/Raman spectroscopy, UV–Vis absorption, and photoluminescence measurements. DFT and TD-DFT calculations were also carried out to support the experimental results. The X-ray analysis revealed significant structural differences: 2PA5N6MP adopted an almost planar conformation (pyridine–phenyl dihedral ~3°), whereas 2PA5N4MP was markedly twisted (~45°), leading to distinct hydrogen-bonding motifs (N–H⋯N dimers vs. N–H⋯O interactions). These geometric disparities influenced their electronic properties: 2PA5N6MP exhibited a narrower HOMO–LUMO gap (≈2.45 eV) than 2PA5N4MP (≈3.77 eV), which was consistent with a pronounced bathochromic shift in absorption. Both isomers showed broad UV–Vis absorption (200–520 nm), but the 6-methyl derivative featured an additional low-energy charge–transfer band around 460 nm (with a maximum at ~500 nm) compared to ~355 nm in the 4-methyl isomer. Likewise, their photoluminescence spectra differed as follows: 2PA5N4MP emitted in the violet–blue region (bands at ~415 and 450 nm), whereas 2PA5N6MP had an extra orange band peaking at ~560 nm (in addition to a ~450 nm band). The red-shifted 560 nm emission of 2PA5N6MP was attributed to intersystem crossing into triplet states, in line with TD-DFT predictions. Furthermore, both isomers readily formed complexes with Eu^3+^ ions, and the Eu^3+^ chelates exhibited the characteristic red f–f emissions (^5^D_0_ → ^7^F transitions ~590–700 nm), demonstrating efficient sensitization of Eu^3+^ luminescence. Overall, the position of the methyl substituent strongly modulates the compounds’ optical behavior, and these isomers show promise as tunable organic dyes and effective ligands for luminescent lanthanide complexes.

## 1. Introduction

Pyridine derivatives represent one of the most versatile classes of heterocyclic compounds, widely explored for their biological, catalytic, and material-oriented properties. The presence of a nitrogen atom within the aromatic ring introduces both electronic asymmetry and coordination capability, making pyridines attractive platforms for designing optically active and multifunctional molecules. In particular, donor–acceptor systems based on amino- and nitro-substituted pyridines exhibit pronounced intramolecular charge–transfer (ICT) effects, high polarizability, and distinct optical responses, which make them promising candidates for organic dyes, luminescent materials, and chemical sensors [1,2,3,4,5,6,7,8,9,10,11,12,13,14,15,16,17,18,19,20,21,22,23,24,25,26,27,28,29,30,31].

Modern design of optically active materials relies on precise control over conjugation, molecular planarity, and the energetic alignment of frontier orbitals. Substituent engineering within heteroaromatic frameworks allows fine-tuning of HOMO–LUMO gaps and thus targeted modulation of absorption and emission profiles. Recent studies have demonstrated how such molecular strategies enable strong red shifts and enhanced optical activity. For example, Liu et al. introduced B–N Lewis pair units into polyaromatic frameworks, achieving a substantial reduction in the LUMO energy and intense near-infrared (NIR) absorption beyond 700 nm [32]. Likewise, Qi et al. developed an oxygen-promoted 6-endo-trig cyclization of β,γ-unsaturated hydrazones with aryl diazonium salts, yielding new pyridazinone and oxazinone heterocycles under mild, metal-free conditions [33]. Both examples illustrate the current trend toward molecular-level tailoring of π-conjugated systems to obtain chromophores with tunable electronic and photophysical properties [34,35,36,37,38,39,40,41,42,43,44,45,46].

Against this background, the present study reports two novel isomers: 2-phenylamino-5-nitro-4-methylpyridine (2PA5N4MP) and 2-phenylamino-5-nitro-6-methylpyridine (2PA5N6MP). Their molecular design follows a push–pull concept, combining an anilino donor and a nitro acceptor on the pyridine core, while the methyl group position modulates conjugation and dipole moment. Comprehensive characterization using X-ray diffraction, IR/Raman, UV–Vis, and fluorescence spectroscopy, supported by DFT calculations, reveals how subtle positional isomerism affects geometry, packing, and photophysical behavior. The strong coloration and stable solid-state luminescence suggest potential applications as tunable organic dyes or optical coating components, while the combination of donor and acceptor coordination sites makes them promising sensitizing ligands for lanthanide ions. These isomers therefore exemplify a modern approach to the molecular design of small, multifunctional organic materials for advanced photonic, sensing, and luminescent applications [47,48,49,50,51,52,53,54,55,56,57,58]. This paper is a continuation of our previous work on amino-pyridine derivatives [59,60,61,62,63,64,65,66,67,68].

## 2. Results and Discussion

### 2.1. XRD Studies of the Studied Isomers

**Figure 1:**
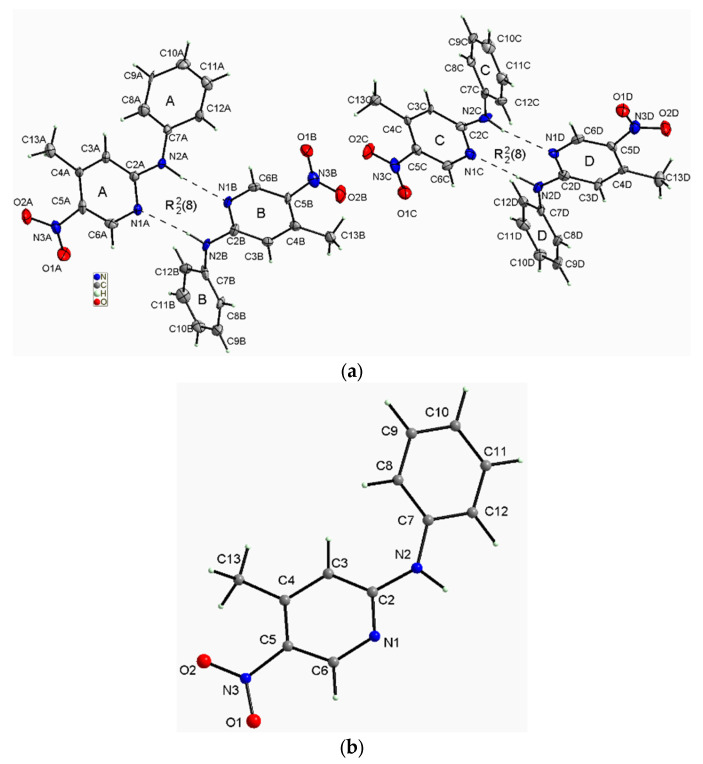
View of the X-ray structure of 2PA5N4MP (**a**) and DFT optimized molecule (**b**). The displacement ellipsoids are shown at the 50% probability level, H atoms with arbitrary radii. The numbering scheme of the corresponding atoms is the same for all molecules (A, B, C, and D).

**Figure 2:**
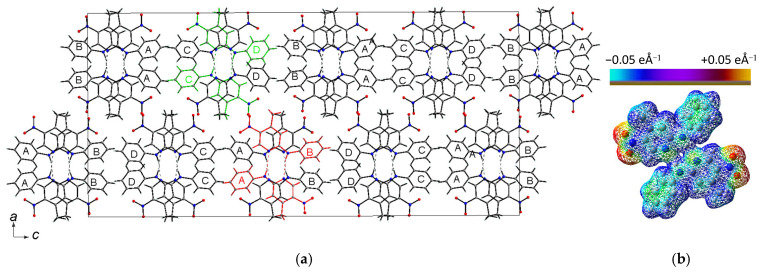
Arrangement of N—H∙∙∙N hydrogen-bonded dimers of PA5N4MP (AB and CD dimers are marked in green and red) in the unit cell along *b*-axis (**a**) and 3D MESP map for dimer (**b**).

**Figure 3:**
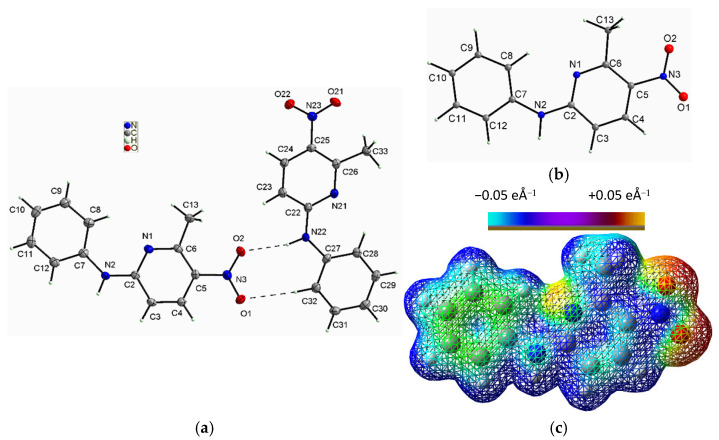
View of the X-ray structure of 2PA5N6MP (**a**), DFT optimized molecule (**b**), and 3D MESP map (**c**).

**Figure 4:**
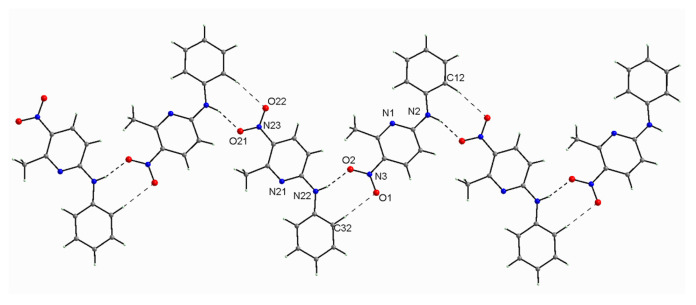
View of hydrogen-bonded chains of 2PA5N6M.

**Figure 5:**
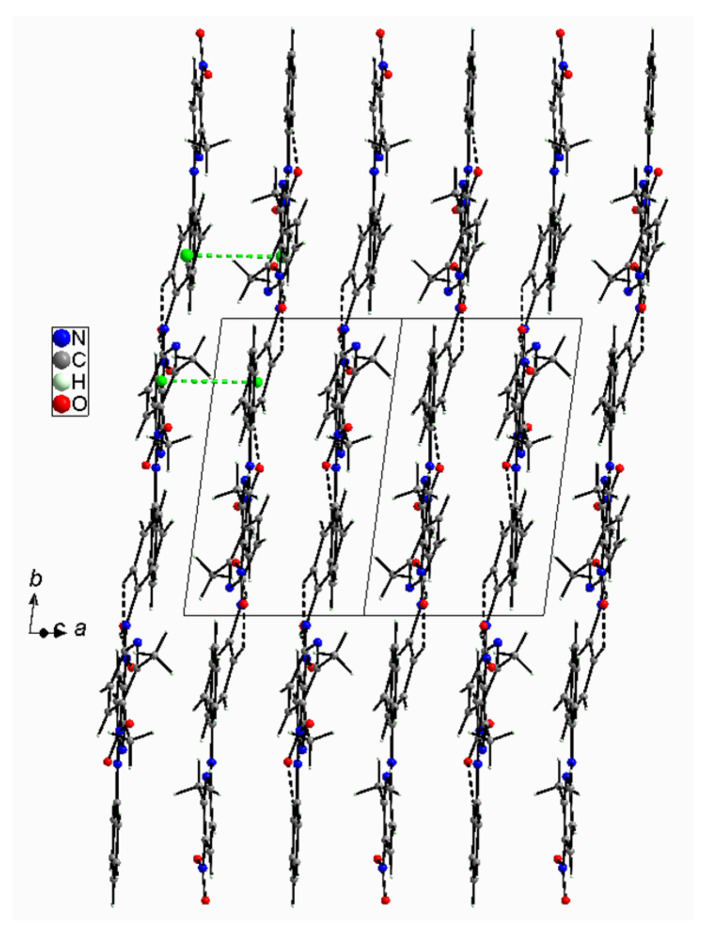
Packing diagram of 2PA5N6MP. The dashed green lines indicate the π···π stacking interactions between the aromatic rings, with centroid–centroid distances of approximately 3.574 Å. The green circles denote the centroids of the aromatic rings.

The crystal and molecular structures of those two studied isomers are shown in Figure 1, Figure 2, Figure 3, Figure 4 and Figure 5. They prove that the substitution position of the nitro and methyl groups strongly influences the structural and optical properties of these derivatives. The orange and yellow colors are characteristic for the isomers, with substitution of the nitro group at position 5 of the pyridine ring. Also, the crystallographic structures of these isomers change depending on the substitution position of both functional groups.

#### 2.1.1. Description of the Structure of 2-*N*-Phenylamino-5-nitro-4-methylpyridine (2PA5N4MP)

2-*N*-phenylamino-5-nitro-4-methylopyridine (2PA5N4MP) crystallizes in the centrosymmetric space group *Pbca* of the orthorhombic system with 32 molecules per unit cell. Its asymmetric unit contains four 2-*N*-phenylamino-5-nitro-4-methylpyridine molecules that form two symmetrically independent molecules linked by N—H∙∙∙N hydrogen bonds with a $R_{2}^{2}$(8) graph dimer, as shown in Figure 3. The geometry of the N—H∙∙∙N hydrogen bonds in the dimers are listed in Table 1.

It should be noted that the centers of the $R_{2}^{2}$(8) graph of both N—H∙∙∙N hydrogen-bonded dimers of asymmetric unit are not the inversion crystallographic centers. Although the asymmetric unit contains four molecules bound in dimers, the geometrical parameters of the respective bonds of the molecules do not differ significantly (Table 1); however, the conformations of the respective molecules (A, B, C, and D) as well as the conformations of both independent dimers are slightly different. All A, B, C, and D molecules exhibit non-planar conformations. The planar NO_2_ group is twisted with respect to the planar pyridine ring in all molecules. The twisting angle ranges from 5.6 (3)° for molecule C to 21.9 (3)° for molecule B. The dihedral angles between the average plane of the pyridine ring and the average plane of the phenyl ring range from 35.7 (3)° for molecule C to 50.3 (3)° for molecule B (Table 1).

In the crystal, the molecules form dimers with N—H∙∙∙N hydrogen bonds; therefore, the DFT calculations were also performed for such a dimer. Although in the crystal both hydrogen-bonded dimers (AB and CD in Figure 3) are independent and slightly different from each other, after optimization they have an inversion center located in the middle of the $R_{2}^{2}$(8) graph as shown in Figure S2b in the Supplementary Materials. When comparing the detailed DFT parameters of the dimer (Table S2 in Supplementary Materials) with those of the monomer (Table 2), it should be stated that the nitro groups are almost coplanar with the pyridine rings (1.20°) and in the monomer the NO_2_ group is more twisted (15.80°), while the dihedral angle between the pyridine and phenyl rings is slightly larger in the dimer (53.60°) than in the monomer (44.70°), which can be seen in Tables S1 and S2 in the Supplementary Materials. The DFT-optimized geometry of the N—H∙∙∙N hydrogen bonds in the dimer in the gas phase, as can be seen from Table 1, does not differ significantly from those in the crystal.

In crystal 2PA5N4MP, four independent molecules are linked by N—H∙∙∙N hydrogen bonds, forming two independent dimers with the $R_{2}^{2}$(8) graph. The dimers are formed from molecules A and B as well as C and D in the crystal, and are related by the b-plane perpendicular to the a-axis and with a translation along the *b*-axis, forming two independent stacks along the *b*-axis that is constructed only of AB dimers or only of CD dimers as shown in Figure 2. There are no directional inter-stack interactions such as hydrogen bonds; however, other interactions such as var der Waals forces and electrostatic interactions hold the dimers into a crystal, forming a three-dimensional architecture (Figure 2). A negative EP value occurs mainly around oxygen atoms, while a positive EP value is associated with hydrogen atoms (Figure 3c), hence the interaction between such dimer units and their further organization takes place between fragments differing in the sign of EP.

#### 2.1.2. Description of the Structure of 2-*N*-Phenylamino-5-nitro-6-methylpyridine (2PA5N6MP)

2-*N*-phenylamino-5-nitro-6-methylpyridine (2PA5N6MP) crystallizes in the centrosymmetric space group of the triclinic system with four molecules per unit cell. Its asymmetric unit contains two 2-*N*-phenylamino-5-nitro-6-methylpyridine molecules linked together via weak N—H∙∙∙O hydrogen bonds with donor–acceptor contact of 2.962 (2) Å. Additionally, both independent molecules are oriented almost coplanar with the pyridine ring of one molecule and the phenyl ring of the other, which enables additional C—H∙∙∙O interactions stabilizing the dimeric structure with a graph of $R_{2}^{2}$(8), as shown in Figure 3. However, DFT optimization of the geometry of such a dimer has not converged; therefore, the DFT optimization was performed only for a monomer (Figure 3b). The DFT conformation of the molecule is closely planar, and the bond distances and angles are in good agreement with the X-ray structure (Table 2. Full details of the DFT optimized geometry of 2-phenylamino-5-nitro-6-methylpyridine molecule are listed in Table S3 (in Supplementary Materials). The three-dimensional molecular electrostatic potential for the 2PA5N6MP molecule was also calculated (Figure 3c). It clearly indicates a negative EP sign around the oxygen atoms of the NO_2_ and near the pyridine ring nitrogen, with a positive EP sign near the hydrogen atoms. Such distribution of EP in the molecule explains the formation of dimers in the crystal which, via translation, form zigzag chains (Figure 4).

This distribution of EP in the molecule explains the formation of dimers in the crystal, which form zigzag chains through translation (Figure 4). These chains in the crystal are aligned to the (101) plane (Figure 5). Adjacent chains interact via π-π between the benzene rings of one chain and the pyridine rings of the neighboring chain. Such arrangement of chains in the crystal is understandable due to the influence of the electron-withdrawing NO2 (EWG) group in the pyridine ring, which is characterized by a significantly larger area of positive sign of EP than the benzene ring without the influence of such an electron-withdrawing group (see Figure 3c and Figure 5 and Table 3). The distance between the centroids of these rings (~3.574 Å) indicates weak interactions between the π-clouds of the rings. These interactions stabilize the structure.

The observed color difference between the two compounds arises from their distinct HOMO–LUMO gaps. A smaller gap corresponds to lower-energy electronic transitions and hence to absorption at longer wavelengths, resulting in an orange appearance, whereas a larger gap shifts absorption toward higher energies, giving a yellow color. Both molecular frameworks also contain polar, hydrogen-bonding groups that enhance solubility in polar media. Indeed, these features ensure very good solubility of the compounds in ethanol and even in water, despite their extended aromatic cores. The presence of ordered π–π stacking (ca. 3.57 Å) in the crystal structure provides additional stabilization and may be advantageous for material applications, combining high solubility with predictable packing favorable for processing and device fabrication.

### 2.2. IR and Raman Spectra

**Figure 6:**
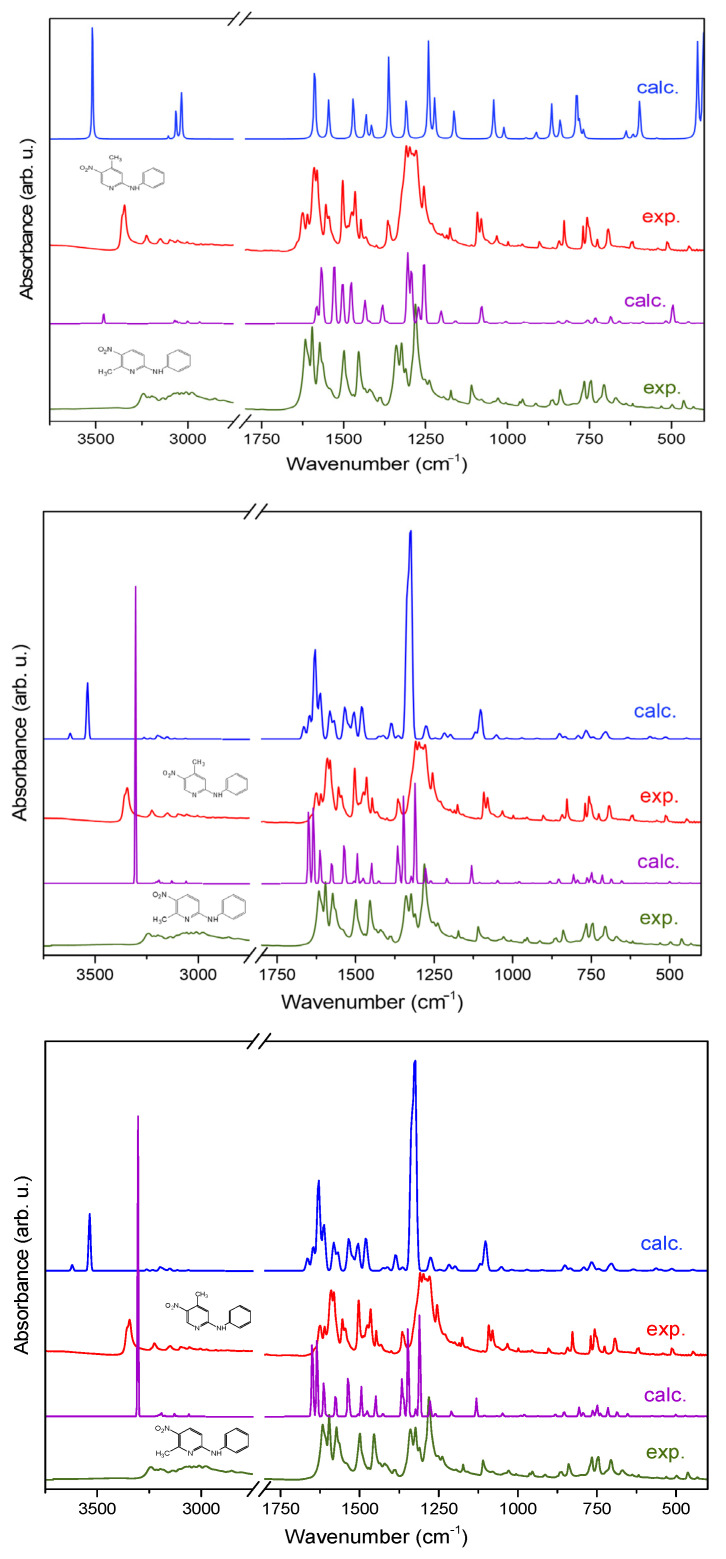
Experimental and calculated IR and Raman spectra of the studied phenyl-aminopyridines in the range of 3500–100 cm^−1^.

IR and Raman spectra of the studied compounds are shown in Figure 6. The X-ray data obtained for both isomers reveal that their structures are substantially different, although they consist of the same units: phenyl and pyridine rings, nitro and methyl groups, and amino bridge. 2PA5N4MP isomer crystallizes in the orthorhombic space group *Pbca*, D2h15 (Z = 32), whereas 2PA5N6MP is triclinic P$\bar{1}$, C11 (Z= 4). The asymmetric unit of the former derivative contains four 2-*N*-phenylamino-5-nitro-4-methylpyridine molecules that form two symmetrically independent dimers linked by N—H∙∙∙N hydrogen bonds (Figure 1). The asymmetric unit of the later isomer contains two 2-phenylamino-5-nitro-6-methylpyridine molecules linked together by weak N—H∙∙∙O hydrogen bonds and additionally, both independent molecules are oriented almost coplanar with the pyridine ring of one molecule and the phenyl ring of the other, which enables additional C—H∙∙∙O interactions that stabilize the dimeric structure, as shown in Figure 3a. The differences between both structures can be seen by comparing their selected geometric parameters (Table 4) as well as the hydrogen bond arrangement presented in Table 5.

In the IR and Raman spectra, the N–H stretching vibration of the phenylamino group is observed at a significantly lower frequency than the free-NH value. For example, 2PA5N4MP shows a strong IR N–H band at ~3344 cm^−1^ (and a corresponding Raman band at ~3547 cm^−1^), while 2PA5N6MP shows the N–H stretch near ~3302 cm^−1^ (IR) and ~3536 cm^−1^ (Raman). These frequencies are downshifted relative to the ~3420 cm^−1^ band expected for a non-hydrogen-bonded secondary aromatic amine. Such red shifts (and often band broadening) indicate that the N–H groups are engaged in hydrogen bonding. Indeed, X-ray analysis confirms that 2PA5N4MP forms strong inter-molecular N–H···N bonds and 2PA5N6MP forms weaker N–H···O bonds in the crystal. Thus, the observed N–H stretching bands reflect the formation of hydrogen-bonded dimers/aggregates in the solid state, which helps stabilize the crystal packing and inter-molecular interactions.

The C_12_H_11_N_3_O_2_ monomeric molecule of the C1 symmetry is the basic unit of the studied isomers. Its 28 atoms give rise to 84 degrees of freedom that contain 3 translational modes, 3 rotational modes, and 78 internal modes. The internal modes could be further subdivided into 28 stretching and 50 bending nodes. For the dimers, the number of the normal vibrational modes is doubled.

The IR features of both isomers are consistent with those reported for structurally related aromatic donor–acceptor systems. In our spectra, the broad N–H stretching band appears near 3300 cm^−1^, as expected for secondary aromatic amines engaged in hydrogen bonding (see Table 5). The diagnostic nitro groups give a clear asymmetric ν(N–O) band at ca. 1535–1555 cm^−1^ and a symmetric ν(N–O) band at ca. 1330–1350 cm^−1^, which falls within the usual ranges reported for nitro-substituted π-systems. The aromatic skeletal region (1600–1480 cm^−1^) contains overlapping C=C/C=N and ν_as(NO_2_) contributions; bands in this window are routinely used to monitor conjugation and donor–acceptor interactions in related systems, including B–N Lewis pair-functionalized anthracenes described by Liu [69], heterocyclic 4H-imidazo-oxadiazin-4-one frameworks reported by Jiang et al. [70], ternary lanthanum coordination polymers based on 8-hydroxyquinoline studied by Zhang et al. [71], and triphenylphosphonium-functionalized PEG nanocarriers investigated by Li et al. [72]. In particular, Zhang et al. observed a heteroaromatic C=N stretching band of 8-hydroxyquinoline at 1579 cm^−1^, which shifts to 1591 cm^−1^ upon coordination to La(III), while Li et al. reported an amide C=O band at ca. 1653 cm^−1^ together with characteristic aromatic TPP bands at 755 and 690 cm^−1^. These examples show that the ν(N–H), ν_as/ν_s(NO_2_) and aromatic C=C/C=N bands of 2PA5N4MP and 2PA5N6MP lie in the expected ranges for functionalized aromatic donor–acceptor frameworks. The small shifts observed between the two isomers therefore confirm the proposed band assignments and indicate that the methyl substituent has only a modest influence on the overall vibrational pattern.

### 2.3. Electron Reflectance and Emission Spectra

**Figure 7:**
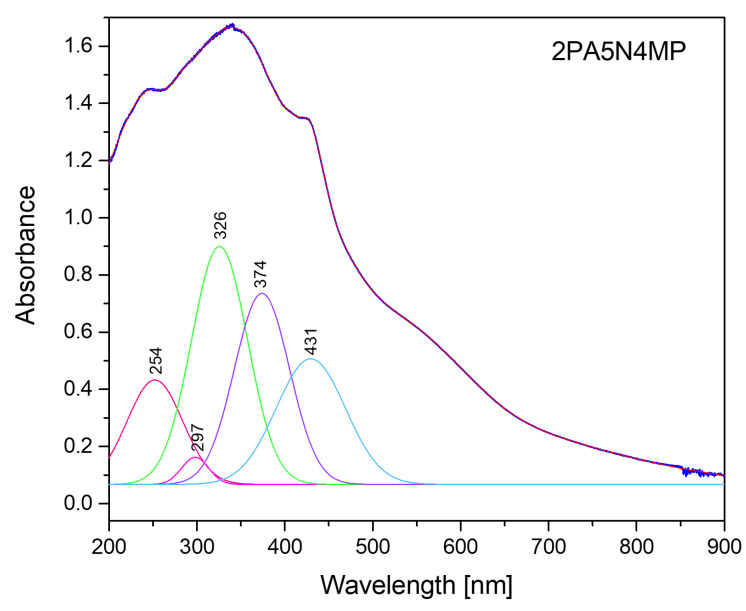

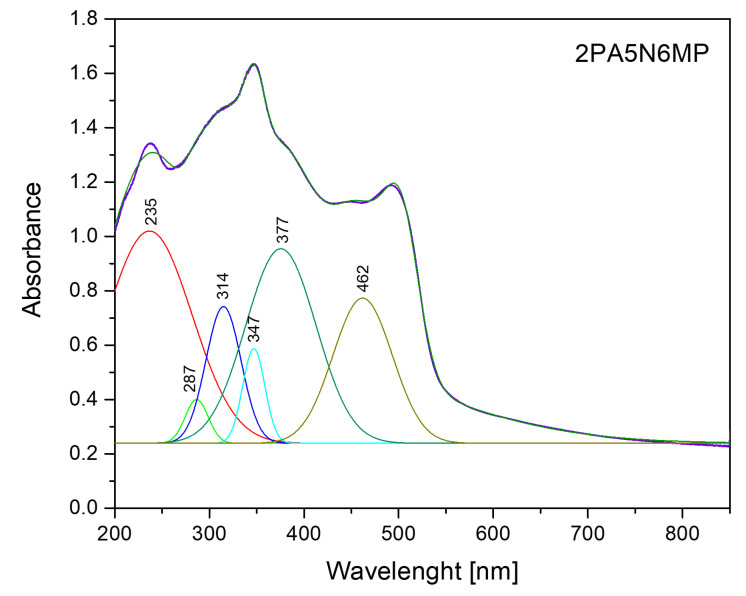
UV–Vis electronic reflectance spectra of 2PA5N4MP and 2PA5N6MP recorded in chloroform. The experimental spectra (black solid lines) were deconvoluted into Gaussian components (colored curves) corresponding to individual electronic transitions.

The electronic reflectance spectra of the studied compounds are presented in Figure 7. They consist of broad absorption bands in the 200–520 nm range, corresponding to electronic transitions characteristic of pyridine-based systems reported previously [73,74,75,76,77,78,79,80,81]. Both spectra were deconvoluted into Gaussian components to visualize the contributions of individual electronic transitions.

For the 2PA5N4MP isomer, the resolved components appear at 254 nm (39,370 cm^−1^), 297 nm (33,670 cm^−1^), 326 nm (30,670 cm^−1^), 374 nm (26,740 cm^−1^), and 431 nm (23,200 cm^−1^). These transitions correspond to π → π* and n → π* excitations within the pyridine framework and between the nitro and amino groups. The 2PA5N6MP derivative exhibits a similar spectral contour, with partially shifted maxima at 235 nm (42,550 cm^−1^), 287 nm (34,840 cm^−1^), 314 nm (31,850 cm^−1^), 347 nm (28,820 cm^−1^), 377 nm (26,530 cm^−1^), and 462 nm (21,650 cm^−1^). The high-energy bands (200–300 nm) correspond to π → π* transitions within the aromatic core, whereas the broad, low-energy absorption near 460 nm originates from charge–transfer interactions between the donor (–NH–Ph) and acceptor (–NO_2_) fragments.

The results of the DFT and TD-DFT calculations carried out for both derivatives are summarized in Table 6 and Table 7, where the energies of the singlet and triplet excited states and their oscillator strengths (f) are listed. The series of experimental bands observed in the 200–520 nm region should thus be assigned to transitions between the singlet states, primarily of π → π* character. These assignments are consistent with the HOMO → LUMO energy gap values obtained from DFT calculations: 3.771 eV (329 nm) for the 2PA5N4MP isomer and 2.454 eV (505 nm) for the 2PA5N6MP isomer. The atomic composition of the molecular orbitals in both derivatives resembles that observed for other amino-methyl-nitro-pyridine systems [59,60,62,79,80]. The HOMO orbital is mainly localized over the amine nitrogen and pyridine C–C/C–N bonds, whereas the LUMO is distributed over the nitro group of the pyridine ring. The theoretical HOMO–LUMO separations correlate well with the strong and broad experimental bands observed in the 200–600 nm region, with maxima near 355 nm for 2PA5N4MP and 500 nm for 2PA5N6MP (see Figure 7).

**Figure 8:**
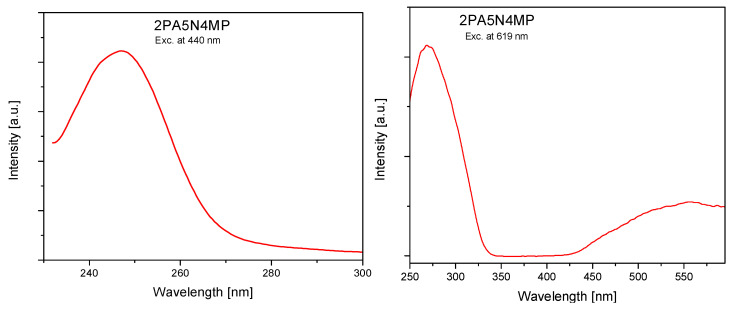

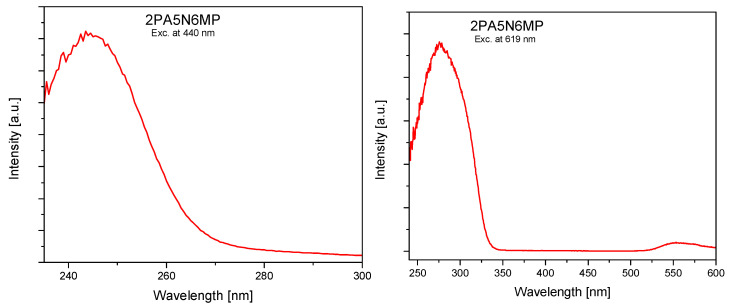
The excitation spectra of the studied isomers, determined at 440 nm and 619 nm.

The excitation spectra of the studied isomers determined at 440 nm and 619 nm are shown in Figure 8. They exhibit strong and broad contours in the 240–350 nm range, as well as a weak and very broad band observed at 400–600 nm in the spectra, determined at 619 nm. The position of components of these contours coincides with the bands observed in the UV–Vis spectra. They derive from the transitions between the singlet electron states of the studied isomers.

**Figure 9:**
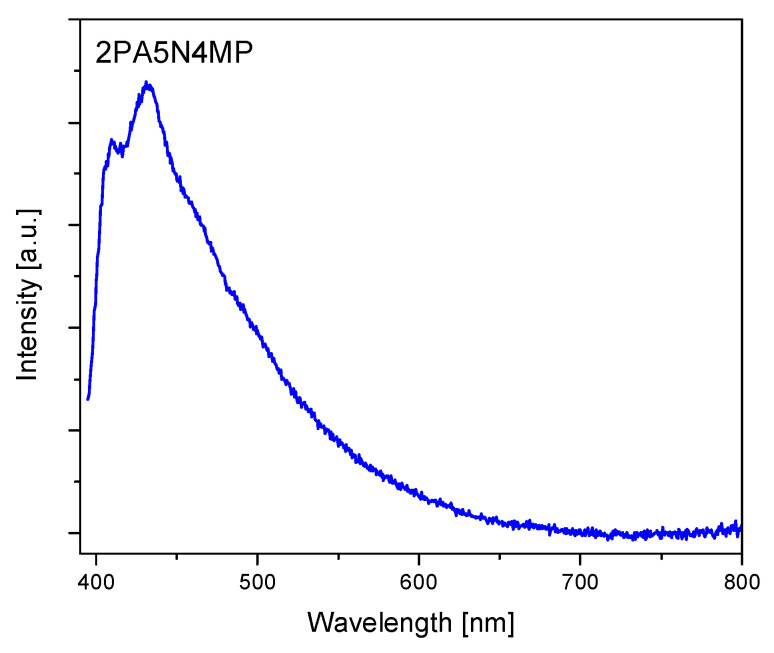
Emission spectrum measured for 2PA5N4MP isomer under excitation at 350 nm.

**Figure 10:**
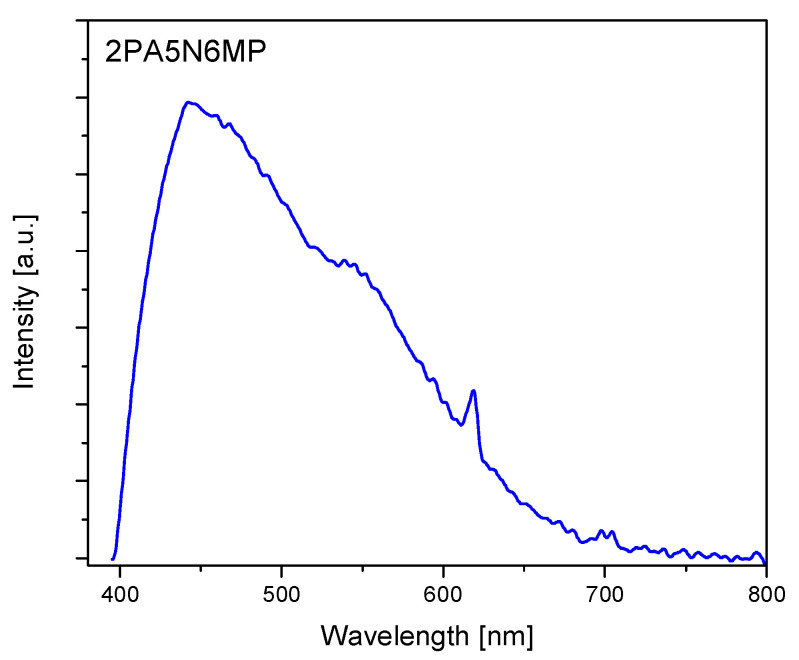
Emission spectrum measured for 2PA5N6MP isomer under excitation at 350 nm.

The emission spectra of the studied derivatives are shown in Figure 9 and Figure 10.

The emission spectrum of the 2PA5N4NP isomer consists of the bands at 415 and 450 nm, and for the 2PA5N6MP isomer, they appear at 450 and 560 nm. They significantly shift to red in comparison with electron absorption transitions observed at about 350 nm (28,571 cm^−1^). Such a notable red shift 4200–8000 cm^−1^ could not be explained as the Stokes shift but is the result of the intersystem crossing process. Considering the experimental energies of the electron levels and the values calculated for the triplet states, the depopulation mechanism of the excited states can be proposed. It is based on the intersystem crossing process, which is a part of a Jablonsky diagram often used to explain the origin of the phosphorescence observed for organic compounds. It contains the following stages: S_0_ ⟶ S_m_ absorption transition; next, the intersystem crossing to the triplet states; and finally, the transition T_1_,T_2_ ⟶ S_0_ is observed in the emission spectra. Room temperature phosphorescence is generally not observed from excited triplet states; however, keeping in mind the calculated energies of the Molecular Orbitals (Table 8), such an explanation of the nature of these transitions is reasonable.

### 2.4. NMR Spectra

**Figure 11:**
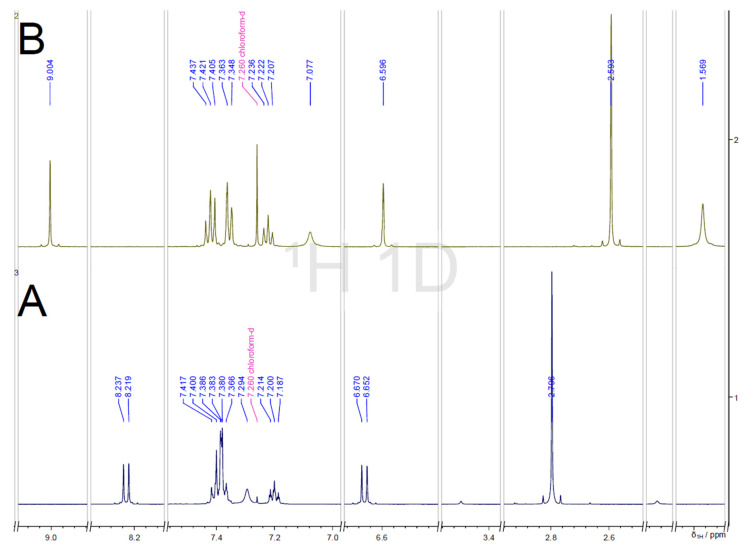
^1^H NMR spectra of the studied isomers: (**A**) 2-*N*-phenylamino-6-methyl-5-nitro- and (**B**) 2-*N*-pheny lamino-4-methyl-5-nitro-pyridines.

**Figure 12:**
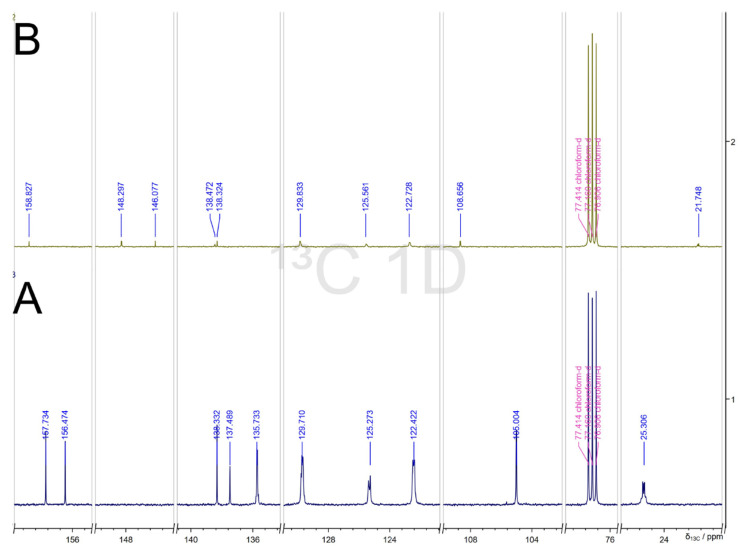
^13^C NMR spectra of the studied isomers: (**A**) 2-*N*-phenylamino-6-methyl-5-nitro- and (**B**) 2-*N*-pheny lamino-4-methyl-5-nitro-pyridines.

The ^1^H and ^13^C NMR spectra of the studied compounds are shown in Figure 11 and Figure 12 respectively (an extended version of Figure 11—Figure S1 is provided in the Supplementary File).

Considering the chemical formula of the studied compounds (C_12_H_11_N_3_O_2_), the expected number of the ^1^H NMR signals can be evaluated. In both isomers, seven characteristic signals are observed, which correspond to the protons of the pyridine and phenyl rings, the methyl group, and the NH chromophore. The aromatic resonances partially overlap in the 7.1–7.4 ppm range, which reduces the number of clearly distinguishable lines compared to the total number of chemically non-equivalent protons. However, the spectra of both compounds differ due to their distinct molecular structures and hydrogen-bonding motifs.

The 2PA5N6MP isomer forms dimeric units stabilized by two inter-molecular N–H···N hydrogen bonds. Its pyridine protons are observed at 9.004, 7.437, 7.421, and 7.405 ppm, while the phenyl protons resonate in the range of 7.386–7.187 ppm. The methyl protons appear at 2.698 ppm. In contrast, the 2PA5N4MP isomer exhibits hydrogen bonds involving the nitro group, a C–H bond, and the pyridine nitrogen atom (N–O···N and O···H–C interactions). Its pyridine protons are found at 8.237 and 8.219 ppm, the phenyl protons at 7.417–7.187 ppm, and the methyl group gives a distinct resonance at 2.846 ppm.

The NH protons, which are particularly sensitive to hydrogen bonding, give rise to signals at 6.596 ppm for the 2PA5N4MP and at 6.670 and 6.652 ppm for the 2PA5N6MP isomer. The partial duplication and broadening of the aromatic signals confirm the dimeric structures observed in the crystal state and indicate the influence of hydrogen bonding on the deshielding of the NH proton.

Similar analysis can be carried out for the carbon atoms present in the studied molecules. For these isomers, five chemical shifts are expected for the atoms constituting the pyridine ring, one of the methyl groups, and six of the phenyl rings. In fact, 12 lines are observed for both isomers. For the 2PA5N4MP isomer, the chemical shifts related to the carbon atoms of the pyridine ring appear at 158.827, 148.297, 138.472, 138.324, and 129.833 ppm, but for the 2PA5N6MP, they appear at 157.734, 156.474, 135.733, 129.710, and 125.150 ppm. On the other hand, the shifts of the phenyl carbon atoms are observed at 146.077, 125.561, 125.140, 122.728, 122.635, and 108.656 ppm for the 2PA5N4MP isomer and at 138.332, 137.489, 125.150, 125.273, 122.422, and 106.004 ppm for the PA5N6MP isomer. The line at 21.748 ppm corresponds to the carbon atom of the methyl chromophore in the 2PA5N4MP derivative and at 25.306 ppm in the 2PA5N6NP isomer. The proposed assignment of the chemical shifts observed for the studied derivatives fit well to the data reported earlier for the similar compounds [81,82,83,84,85,86].

The dimeric structure stabilized by O–H···N and C–H···O hydrogen bonds was further examined using DFT/GIAO calculations (PCM, CHCl_3_). The calculated deshielding of the N–H proton (≈9 ppm) compared to the experimental value (≈6.6 ppm) supports the presence of inter-molecular hydrogen bonding and suggests that such interactions may partially persist in solution, although our structural conclusions refer primarily to the crystalline state.

In the case of the 6-methyl derivative, the dimer stabilized by an inter-molecular N–H···N hydrogen bond shows similar behavior. DFT/GIAO calculations (PCM, CHCl_3_) revealed strong deshielding of the N–H proton (≈9 ppm), consistent with the experimentally observed chemical shift (~6.6 ppm) and with the presence of a robust N–H···N interaction in the solid state.

### 2.5. Prospective Applications of the Studied Isomers

The color of the studied isomers allows them to be used as potential dyes in the production of plastic foils for food packaging. At the same time, their spectroscopic and structural properties indicate a much broader applicability as multifunctional components in optical and photonic materials. The strong and tunable absorption within the 300–600 nm region, combined with efficient emission in the visible range, suggests that these derivatives can act as color-stable organic chromophores when incorporated into polymer matrices. The formation of hydrogen-bonded dimers in the solid state enhances the rigidity of the crystalline lattice, limits nonradiative deactivation, and ensures photostability, which are key parameters for optical coatings and dye-doped polymer materials.

Moreover, the donor–acceptor architecture of the studied compounds, resulting from the interaction between the –NH–Ph and –NO_2_ groups, allows modulation of their electronic and optical response, opening perspectives for charge–transfer-based emissive and sensing systems. The calculated HOMO–LUMO energy gaps and extended π-conjugation indicate that these molecules can be used in optoelectronic coatings and light-emitting materials. Additionally, their ability to coordinate metal ions, especially lanthanides, provides an opportunity to design new luminescent hybrid systems for advanced photonic applications, including sensors, light-emitting diodes, or functional optical packaging materials. The syntheses were performed using a definite amount of the ligand dissolved in c.a 25 mL methanol and Eu(NO_3_)_3_ dissolved in methanol added dropwise to this solution. The molar ratio of the ligand to europium salt was 2:1. The obtained solutions were stirred in a magnetic mixer and heated at 50 °C for 3 h, up to complete evaporation. The resulting powdered materials were studied using the emission spectrometer described above. The obtained spectra observed under excitation at 310 nm are shown on Figure S3 in the Supplementary File.

The emission of Eu^3+^ in the studied complexes corresponds to ^5^D_0_ ⟶ ^7^F_j_ f-f transitions of europium covering 550–710 nm spectral range. The excitation at 310 nm gives rise to luminescence bands at 595 nm (16,807 cm^−1^), 615 (16,260), 620 (16,129), 652 (15,337), and 699 nm (14,205 cm^−1^) which correspond to the transitions from ^5^D_0_ of Eu^3+^ excited states to ^7^F_0,1,2,3,4_ multiplets, respectively. Additionally, low-intense emission bands located at 593 (16,863), 610 (16,393), and 704 nm (14,205 cm^−1^) are associated with the transition from the ^5^D_1_ level to the respective ^7^F_j_ excited levels. The obtained results confirm that the phenylamino–pyridine ligands easily participate in chelation of the lanthanide ions.

## 3. Materials and Methods

### 3.1. Synthesis

**Scheme 1:**
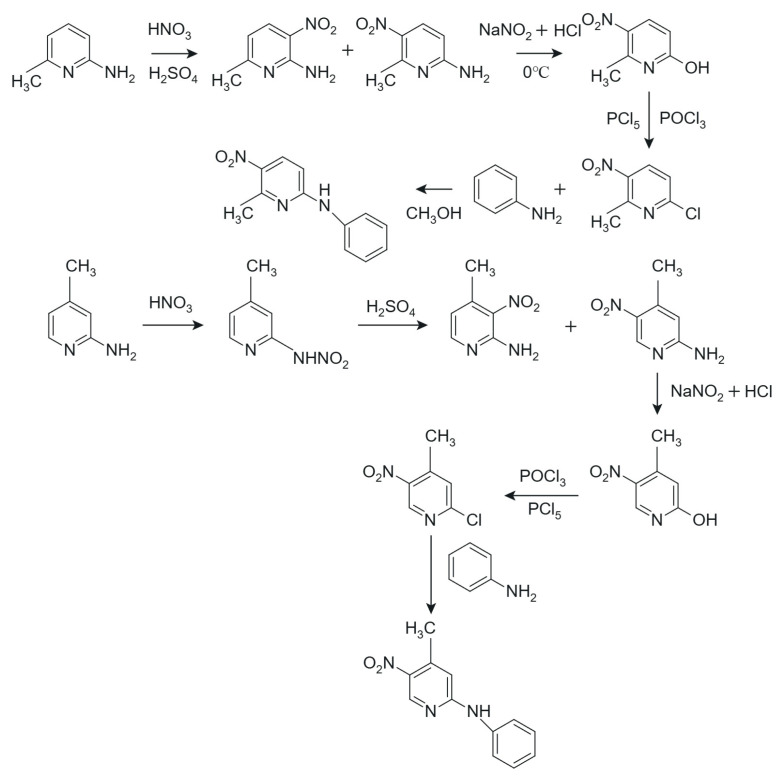
Synthetic route to obtain 2PA5N4MP and 2PA5N6MP.

Two 2-*N*-phenylamino-(4 or 6)-methyl-5-nitropyridine isomers were synthesized, modifying the method described in our earlier work [87]. They were synthesized starting from 2-amino-3 or 6-methylpyridine. It was nitrated and transformed into 2-hydroxy-forms in the first step, and to 2-chloro-derivatives using POCl_3_ as the substrate [87]. The final compounds were obtained in the reaction with phenylamine. The synthetic route to obtain 2-*N*-phenylamino-5-nitro-4-methylopyridine (2PA5N4MP) and 2-*N*-phenylamino-5-nitro-6-methylpyridine (2PA5N6MP) is shown in Scheme 1. Multiple recrystallizations of these compounds from ethanol solution were performed to obtain yellow (2PA5N4MP) or orange (2PA5N6MP) crystals suitable for X-ray single-crystal studies. The chemical composition was established by microanalysis and was in good agreement with theoretical stoichiometry.

Analysis of yellow crystals 2PA5N4MP: Found: and in parathesis calculated for C_12_H_11_N_3_O_2_: C 62.87 (62.82%), H 4.84 (4.81%), and N 18.33 (18.29%), melting point 360 K. Analysis of orange crystals 2PA5N6MP: Found: and in parathesis calculated for C_12_H_11_N_3_O_2_: C 62.87 (62.55%), H 4.84 (4.88%), N 18.33 (18.12%), M_p_ 405 K.

### 3.2. Single-Crystal X-Ray Diffraction

Crystals suitable for single-crystal X-ray diffraction analysis was obtained by recrystallization from a methanol/ethanol diffusion solvent system. The X-ray intensity data for crystals 2PA5N4MP and 2PA5N6MP were collected using graphite monochromatic MoK_α_ radiation on a four-circle κ geometry Xcalibur diffractometer with Sapphire2 area CCD detector (Rigaku Polska Sp. z o.o., Wroclaw, Poland). Data collections were made using the CrysAlis CCD program [88] (Rigaku Polska Sp. z o.o., Wroclaw, Poland). Integration, scaling of the reflections, correction for Lorenz and polarization effects and absorption corrections were performed using the CrysAlis Red program [88] (Rigaku Oxford Diffraction Ltd. (Oxfordshire, UK), Version 1.171.35.11 (2011)). The structures were solved by direct methods using SHELXT-2014/7 [89] and refined using SHELXL-2018/3 program [90]. The hydrogen atoms joined to carbon atoms were introduced in their geometrical positions and treated as rigid. The H atoms involved in the hydrogen bonds were refined. The final difference Fourier maps showed no peaks of chemical significance. Details of the data collection parameters, crystallographic data and final agreement parameters are collected in Table 8. Visualizations of the structures were made with the Diamond 3.0 [91].

### 3.3. Infrared and Raman Studies

IR spectra were measured using a usually applied in our studies spectrometer: Nicolet iS50 FT-IR (Thermo Fisher Scientific Inc., Warsaw, Poland) equipped with an Automated Beamsplitter exchange system (iS50 ABX containing DLaTGS KBr detector and DLaTGS Solid Substrate detector for mid-IR and far-IR regions, respectively) (Thermo Fisher Scientific Inc., Warsaw, Poland). Built-in all-reflective diamond ATR module (iS50 ATR), Thermo Scientific Polaris™ (Thermo Fisher Scientific Inc., Warsaw, Poland) and HeNe laser as an IR radiation source. Polycrystalline IR spectra were collected in the 4000–100 cm^−1^ range. The advanced ATR correction software, part of the OMNIC^TM^ 6.2 program attached to Thermo Scientific Nicolet^TM^ FT-IR spectrometer (Thermo Fisher Scientific Inc., Warsaw, Poland), was used in the studies of polycrystalline samples. Spectral resolution 4 cm^−1^ was applied in the measurements.

Raman spectra in the 4000–80 cm^−1^ range were measured in backscattering geometry with an FT Bruker 110/S spectrometer (bruker, Warsaw, Poland). The resolution was 2.0 cm^−1^. The YAG:Nd (excitation wavelength 1064 nm) laser was used as an excitation source.

### 3.4. Electron Absorption Spectra

Room temperature electron absorption spectra were measured in the 200–1500 nm spectral range using a Cary-Varian 5E UV-VIS-near-IR spectrophotometer (SpectrLab Scientific Inc., Warsaw, Poland). In the case of a weak spectrum signal, the spectrophotometer was switched to measurements in the diffuse reflectance mode. Diffuse reflectance spectra were recorded with Praying Mantis diffuse reflectance accessories (Harrick Scientific Products Inc., Warsaw, Poland). In these measurements, the baseline was first recorded for Al_2_O_3_ powder, and next, this line was subtracted from the obtained value for powder sample spectra.

### 3.5. Emission Spectra Measurements

Decay emission spectra were recorded with a grating spectrograph (Princeton Instr. Model Acton 2500i—Teledyne Vision Solutions, Warsaw, Poland) coupled to a streak camera (Hamamatsu Model C5680—Hamamatsu Co., Warsaw, Poland). For excitation, a femtosecond laser (Coherent Model “Libra”—Coherent Inc., Warsaw, Poland) was used. The laser delivers a train of 89 fs pulses at a wavelength of 800 nm and pulse energy of 1 mJ, with repetition rates regulated up to 1 kHz. To attain light pulses at different wavelengths, the laser was coupled to an optical parametric amplifier (Light Conversion Model OPerA—Coherent Inc., Warsaw, Poland) that can operate in the range of 230–2800 nm.

### 3.6. Quantum Chemical Calculations

All DFT calculations were performed in the gas phase without applying any solvation model. The geometry optimization of the molecular structure of the studied compound was carried out for the monomeric unit using Gaussian 09 program package [92]. In the calculations, the atomic positions from X-ray studies were taken as the input data. Only one stable conformer with zero vibrational potential energy was found with such an approach. All the calculations were performed by applying density functional three-parameter hybrid (B3LYP) methods [93,94,95] with the 6-311G(2d,2p) [96,97] basis set, starting from the X-ray geometry. After the geometry optimization the three-dimensional molecular electrostatic potential (3D MESP) maps were calculated. The calculated and experimental values were compared using two scaling factors (see Table 3) to correct the evaluated wavenumbers for vibrational anharmonicity and deficiencies inherent to the used computational level. The IR and Raman wavenumbers were calculated for a single molecule.

The Potential Energy Distribution (PED) of the normal modes among the respective internal coordinates was calculated using the BALGA v 1.0 program (Nowak, M.J., Lapinski, L.), BALGA computer program for PED calculations [98]. Gauss View 4.1 program [99] was used to visually present the HOMO and LUMO diagrams given in this work.

Vector displacements of the atoms from their equilibrium positions during vibration and the pictures of these displacements were prepared using the ChemCraft B802bt program, and it also visualized modes in an animated way [100].

The scaling of the theoretical wavenumbers to compare them with the experimental values was performed using a linear correlation. A 0.965 scaling factor was used and was calculated according to the method reported by Palafox and Rastogi [101]. The theoretical Raman intensities were calculated using the RAINT computer program [102].

## 4. Conclusions

Single-crystal X-ray diffraction shows that 2-phenylamino-5-nitro-4-methylpyridine (2PA5N4MP) forms dimers linked by N–H···N hydrogen bonds, whereas 2-phenylamino-5-nitro-6-methylpyridine (2PA5N6MP) forms N–H···O dimers with additional C–H···O contacts and π–π interactions. Molecular electrostatic potential (MEP) maps assign negative potential to the nitro oxygens and the ring nitrogen and positive potential to the N–H site and adjacent aryl C–H positions; these donor/acceptor sites match the hydrogen-bond motifs identified crystallographically.

IR spectra display the expected N–H stretch near ~3300 cm^−1^ and the NO_2_ stretches (ν_as ≈ 1530–1550 cm^−1^, ν_s ≈ 1330–1350 cm^−1^); aromatic C=C/C=N bands occur at 1600–1480 cm^−1^. Scaled DFT frequencies reproduce the experimental bands and resolve small, position-dependent shifts between the isomers.

TD-DFT yields HOMO–LUMO gaps of ~3.77 eV (2PA5N4MP) and ~2.45 eV (2PA5N6MP), consistent with the UV–Vis profiles (near-UV/blue vs. extended visible absorption). Orbital analysis places the HOMO on the phenylamino/pyridine donor fragment and the LUMO on the nitro-substituted ring, indicating π–π/charge–transfer* lowest transitions. The emission behavior follows this assignment and the expected nonradiative pathways for charge–transfer states.

Overall, methyl substitution at C-4 vs. C-6 controls hydrogen-bond topology, crystal packing, and the optical gap while preserving the donor–acceptor mechanism. The two isomers provide compact, position-tunable push–pull chromophores and practical building blocks for luminescent and sensing materials (including potential antenna ligands for lanthanides).

### Supporting Information

Additional materials containing the DFT-optimized parameters for both isomers 2PA3N4MP and 2PA3N6MP. CCDC No. 2426412 and 2426413 contain the supplementary crystallographic data for 2PA3N4MP and 2PA3N6MP, respectively. These data can be obtained free of charge via https://www.ccdc.cam.ac.uk/structures/ (accessed on 12 September 2025) or from the Cambridge Crystallographic Data Centre, 12 Union Road, Cambridge CB2 1EZ, UK; fax: (+44) 1223-336-033; or e-mail: deposit@ccdc.cam.ac.uk.

## Figures and Tables

**Table 1:** Selected geometrical parameters (Å, °) for PA5N4MP.

		Mol. A	Mol. B	Mol. C	Mol. D	DFT
N3—O1		1.217 (4)	1.231 (4)	1.237 (4)	1.244 (4)	1.229
N3—O2		1.237 (4)	1.233 (4)	1.232 (4)	1.238 (4)	1.230
C5—N3		1.468 (5)	1.466 (5)	1.462 (5)	1.457 (5)	1.458
C2—N2—C7		127.7 (3)	127.4 (3)	131.2 (4)	127.0 (4)	129.98
Dihedral angle between the planes:						
NO_2_ (O1N3O2)/pyridine ring (N1, C2–C6) pyridine ring (N1, C2–C6)/phenyl ring (C7–C12)		20.0 (3)	21.9 (3)	5.6 (3)	11.3 (3)	15.80
		45.1 (3)	50.3 (3)	35.6 (3)	50.2 (3)	44.70
Hydrogen-bond geometry in crystal (Å, °)						
D—H···A	D—H	H···A	D···A		D—H···A	
N2A—H21···N1B	0.96 (4)	2.07 (4)	3.021 (4)		172 (3)	
N2B—H22···N1A	0.85 (2)	2.20 (2)	3.041 (5)		171 (4)	
N2C—H23···N1D	0.85 (4)	2.21 (4)	3.032 (5)		163 (4)	
N2D—H24···N1C	0.91 (4)	2.07 (4)	2.975 (5)		174 (4)	
Hydrogen bonds in dimer in gas phase (Å, °)						
D—H···A	D—H	H···A	D···A		D—H···A	
N2A–H21···N1B	1.029	2.005	3.030		173.97	
N2B–H22···N1A	1.029	2.005	3.030		173.97	

**Table 2:** Selected geometrical parameters (Å,°) for 2PA5N6MP.

		X-Ray		DFT	
N3—O1/N23—O21		1.239 (2)/1.239 (2)		1.242	
N3—O2/N23—O21		1.243 (2)/1.238 (2)		1.226	
C5—N3/C25—N23		1.436 (3)/1.437 (3)		1.454	
C2—N2—C7/C22—N22—C27		131.9 (2)/132.1 (2)		131.64	
Dihedral angle between the planes:					
NO_2_ (O1N3O2)/pyridine ring (N1, C2–C6)		3.50 (5)/2.40 (5) *		0.00	
pyridine ring (N1, C2–C6)/phenyl ring (C7–C12)		2.31 (5)/4.30 (5) *		0.00	
D—H···A		D—H	H···A	D···A	D—H···A
N22—H22···O2	0.87 (2)	2.11 (3)	2.962 (2)	136.4 (13)	X-ray
C32—H32···O1	0.95	2.56	3.506	175	X-ray
N2—H2···O21 *	0.87 (2)	2.16 (2)	2.962 (2)	153	X-ray
C12—H12···O22 *	0.95	2.62	3.511 (3)	157	X-ray
Symmetry code: (*i*) x + 1, y, z − 1.					
N2—H2···O1	1.014	1.814	2.632	135.17	DFT

* for the second independent molecule.

**Table 3:** Selected geometrical parameters (Å, angles in degrees) of the studied derivatives.

	2PA5N4MP	2PA5N6MP
C_ϕ_—NH—C_φ_ bridge		
C2—N2	1.365	1.361
N2—H2	0.987	0.870
N2—C7	1.414	1.407
C2—N2—C7	128.3	123.03
O1—N3—02 nitro group		
C3—N3	1.463	1.436
N3—O1	1.234	1.239
N3—O2	1.232	1.240
O1—N3—O2	123.1	121.71
C4—C13H_3_ methyl group		
C4—C13	1.502	1.501
C_13_—H	0.980	0.980
C_13_—H	0.980	0.980
C_13_—H	0.980	0.980

**Table 4:** Hydrogen-bond geometry (Å, °) in the studied isomers.

*D*—H···*A*	*D*—H	H···*A*	*D*···*A*	*D*—H···*A* [°]
2PA5N4MP (inter-molecular HB)				
N2A—H21····N1B	0.96	2.07	3.020	172
N2B—H22······N1A	0.85	2.20	3.041	171
N2C—H23·····N1D	0.97	2.19	3.032	162
N2D—H24·····N1C	0.91	2.07	2.976	174
2PA5N6MP (inter-molecular HB)				
N22—H22·····O2	0.87	2.11	2.962	165
C31—H31····O1	0.95	2.56	3.506	175
N2—H2······O21′	0.87	2.16	2.962	153
C11—H11····O22′	0/95	2.62	3.511	157

**Table 5:** Experimental and calculated wavenumbers with assignment of the observed bands to respective normal modes obtained for the dimeric molecules.

2PA5N4MP			2PA5N6MP			
Calc.	Exp.		Calc.	Exp.		
IR, RS	IR	RS	IR, RS	IR	RS	Assignment
3613 vw			3547 vs	3344 s		νN-H
3536 s						νN-H
			3302 vs	3224 w		νNHO
3237	3240 m					νsNHN
3215	3213 m					νasNHN
			1650 s	1624 m	1646 vw	δNHO
1657 v		1623 m				δasNHN
1648 m	1615 s	1616 w				δsNHN
1610 m						δsNHN
			1581 w	1580 s	1586 w	ν(NO) + ν(ϕ)
1572 m	1571 s	1597 w				δasNHN
1546 m	1562 sh					ν(NO) + ν(ϕ)
			1549 w	1553 m		ν(NO) + δNHO + δCHO
				1543 m	1543 vw	ν(NO) + δNHO + δCHO
1542 m	1543 sh	1545 w	1536 m			ν(NO) + ν(ϕ)
1500 m						ν(NO) + ν(ϕ)
1499 m	1497 s	1500 w	1490 m	1474 m	1474 w	ν(NO) + ν(ϕ)
1451 m	1433 w	1423 vw				ν(ϕ) + δ(CH_3_) + δasNHN
1444 m						ν(ϕ) + δsNHN
			1364 m	1363 m	1364 w	δ(NHO) + ν(ϕ) + δ(CH_3_)
1338 vs	1338 s	1335 s				δ(NH) + ν(ϕ)
1336 w						δ(NH) + ν(ϕ)
1319 vw	1321 s	1327 vs				ν_s_(NO_2_) + ν(ϕ) + δ(CH_3_)
		1312 s	1309 vs	1306 vs	1308 s	ν_s_(NO_2_) + ν(ϕ) + δ(CH_3_)
			1296 w	1297 vs	1300 s	ν_s_(NO_2_) + ν(ϕ) + δ(CH_3_)
1294 w	1309 m	1293 s				νCN + ν_s_(NO_2_)
				1287 vs		δ(NCNHO)
				1277 vs	1277 vs	δ(NCNHO)
			1251 m	1253 s		δ(NCNHO)
1253 w	1251 m	1257 m				ν(NCN)
1249 w			1245 w	1227 w	1229 w	ν(NCN) + δNH
1234 w	1237 m	1237 w				ν(C-NH)
1231 w		1221 vw				ν(C-NH)
			1221 w		1207 w	ν(CN)_(NH)_
1105 m	1108 m	1108 w				νCN_(NO2)_
			1080 vw	1078 m	1079 w	νCN_(NO2)_
834 w	837 m	836 w	854 vw	840 w	824 w	δ_s_(NO)_ϕ_ + τ(ϕ)
787 w			800 w			δ_s_NHN
			773 w	767 m	756 w	δ(NO_2_) + ρ(CH_3_)
767 w	763 m					δ_s_NHN
736 w						δNCN
	703 m	746 w				ρNHN
			674			δNHO
670 w	667 w					τasNHN
637 w	636 w	635 vw				δCNCN
			224 w		219 w	δCNC
202 w						δ(CNHC)_ϕ+θ_
		135 sh				δ(CNHC)_ϕ+θ_

**Table 6:** Calculated singlet and triplet electron levels of the 2PA5N4MP molecule.

Electron Levels	eV	nm	Oscillator Strength
singlets			
(1)	3.3648	368.47	0.0014
(2)	3.5956	344.82	0.0080
(3)	3.6292	341.63	0.9036
(4)	3.8372	323.11	0.0004
(5)	3.8404	322.84	0.0544
(6)	4.1984	295.31	0.0003
(7)	4.2553	291.36	0.0572
(8)	4.2674	290.54	0.0007
(9)	4.2725	290.19	0.0770
(10)	4.3889	282.49	0.0143
(11)	4.4122	281.01	0.0000
(12)	4.4932	275.94	0.0003
(13)	4.4986	275.60	0.0053
(14)	4.5171	274.48	0.0018
(15)	4.5214	274.22	0.0000
(16)	4.6104	268.93	0.0998
(17)	4.6267	267.98	0.0020
(18)	4.6921	264.24	0.0033
(19)	4.7129	263.08	0.0946
(20)	4.7723	259.80	0.0270
triplets			
(1)	1.6540	749.61	0.0769
(2)	1.8336	676.18	0.0041
(3)	1.9787	626.58	0.2631
(4)	2.0317	610.26	0.0001
(5)	2.0489	605.12	0.2142
(6)	2.1133	586.70	0.0017
(7)	2.2741	545.20	0.0377
(8)	2.3019	538.61	0.0000
(9)	2.3284	532.49	1.7137
(10)	2.3677	523.64	0.0000

**Table 7:** Calculated singlet and triplet electron levels of the 2PA5N6NP molecule.

Electron Levels	eV	nm	Oscillator Strength
singlets			
(1)	1.9553	634.08	0.0005
(2)	3.1721	390.86	0.0293
(3)	3.2259	384.34	0.6634
(4)	3.4036	364.27	0.6654
(5)	3.5239	351.84	0.0001
(6)	3.5842	345.92	0.0004
(7)	3.6283	341.71	0.0011
(8)	3.7496	330.66	0.0006
(9)	3.7520	330.45	0.0001
(10)	3.9468	314.14	0.0026
(11)	4.0681	304.77	0.0042
(12)	4.0948	302.79	0.0006
(13)	4.1003	302.38	0.0031
(14)	4.1303	300.18	0.0910
(15)	4.1933	295.67	0.0047
(16)	4.2553	291.37	0.0169
(17)	4.3948	282.12	0.0026
(18)	4.4714	277.28	0.0000
(19)	4.5383	273.20	0.2150
(20)	4.5518	272.39	0.0022
triplets			
(1)	3.2259	384.34	0.6636
(2)	3.5239	351.84	0.0001
(3)	3.7496	330.66	0.0006
(4)	3.9468	314.14	0.0026
(5)	4.1090	301.74	0.0307
(6)	4.1933	295.67	0.0047
(7)	4.3948	282.12	0.0026
(8)	4.4714	277.28	0.0000
(9)	4.5518	272.39	0.0022
(10)	4.6538	266.41	0.0001

**Table 8:** Crystallographic data for 2PA5N4MP and 2PA5N6MP.

	2PA5N4MP	2PA5N6MP
Empirical formula	C_12_H_11_N_3_O_2_	C_12_H_11_N_3_O_2_
Formula weight (g·mol^–1^)	229.24	229.24
Crystal system, space group	Orthorhombic, *Pbca*	Triclinic, P$\bar{1}$
*a*, (Å)	23.6386 (14)	7.2576 (4)
*b*, (Å)	7.3142 (4)	10.8585 (7)
*c*, (Å)	49.929 (3)	13.7842 (6)
α, β, γ (°)	90, 90, 90	87.188 (4), 87.619 (4), 83.061 (5)
*V* (Å^3^)	8632.6 (9)	1076.34 (10)
*Z*	32	4
*D*_calc_ (mg·cm^–3^)	1.411	1.415
μ (mm^–1^)	0.100	0.100
Crystal size (mm)	0.22 × 0.12 × 0.10	0.23 × 0.12 × 0.10
λ (Å)	Mo *K*α, 0.71073	Mo *K*α, 0.71073
Temperature (K)	100 (2)	100 (2)
θ range (°)	2.4–7.0	2.5–29.3
Absorption correction	multi-scan	multi-scan
*T*_min_/*T*_max_	0.983/1.000	0.975/1.000
Refls measured,	126,761	12,740
independent,	9428	5122
observwed, I > 2σ(I)	4045	3601
*R*_int_	0.099	0.030
Refinement on *F*^2^		
*R*[*F*^2^ > 2σ(*F*^2^)]	0.102	0.058
*wR*(*F*^2^ all reflections) ^a^	0.170	0.129
Goodness-of-fit, *S*	1.01	1.00
Δρ_max_, Δρ_min_ (e Å^–3^)	+0.27, −0.32	+0.33, −0.31

^a^ *w =* 1/[*σ*^2^(*F_o_*^2^) *+* (*aP*)^2^ *+ bP*], where *P* = (*F_o_*^2^ + *2F_c_*^2^)/3. The *a* and *b* parameters are 0.0424 and 0 for 2PA5N4MP and 0.0333 and 1.2084 for 2PA5N6MP.

## Data Availability

The data presented in this study are openly available in Cambridge Crystallographic Data Centre at https://www.ccdc.cam.ac.uk/structures/, reference number CCDC 2426412, 2426413.

## Supplementary Materials

The following supporting information can be downloaded at https://www.mdpi.com/article/10.3390/ijms262311522/s1.
