# Supplementary material for: Structural and Optical Properties of New 2-Phenylamino-5-nitro-4-methylopyridine and 2-Phenylamino-5-nitro-6-methylpyridine Isomers
Source: Int J Mol Sci. 2025 Nov 27;26(23):11522. doi: 10.3390/ijms262311522 (PMC12692667; doi:10.3390/ijms262311522)
Supplement: Supplementary file 1 [file ijms-26-11522-s001.zip › ijms-3979550-supplementary.pdf]

Structural and optical properties of new 2-phenylamino-5-nitro-4-methylopyridine and 2-phenylamino-5-nitro-6-methylpyridine isomers

P. Godlewska [1], J. Janczak [2], R. Lisiecki [2], J. Hanuza [2], P. Ropuszynska-Robak [1], L. Dymińska [1] and W. Sąsiadek [1]

[1] Department of Bioorganic Chemistry, Faculty of Production Engineering, Wroclaw University of Economics and Business, 118-120 Komandorska str., 53-345 Wrocław, Poland
[2] Institute of Low Temperature and Structure Research, 2 Okólna str., 50-422 Wrocław, Poland

Content

Table S1. Optimized parameters (Å,°) for 2-*N*-phenylamino-5-nitro-4-methylopyridine (PA5N4MP - **2**)
Table S2. Optimized parameters (Å,°) for 2-*N*-phenylamino-5-nitro-4-methylopyridine HB dimer (PA5N4MP - **2**)
Table S3. Optimized parameters (Å,°) for 2-phenylamino-5-nitro-6-methylpyridine (PA5N6MP - **1**)
Table S4. Experimental and calculated wavenumbers with assignment of the observed bands to respective normal modes obtained for the dimeric molecules.

Figure S1. 1H NMR spectra of 2-N-pheny lamino-4-methyl-5-nitropyridine.
Figure S2. 1H NMR spectra of 2-N-phenylamino-6-methyl-5-nitropyridine.
Figure S3. The emission spectra of the europium coplexes obtained in the present work

Table S1. Optimized parameters (Å,º) for 2-*N*-phenylamino-5-nitro-4-methylopyridine (PA5N4MP - **2**).

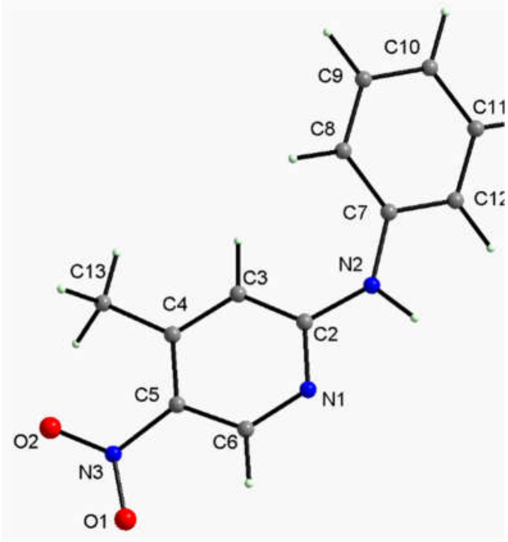

N3–O1	1.229	N3–O2	1.230
N1–C2	1.349	C2–C3	1.408
C3–C4	1.389	C4–C5	1.413
C5–C6	1.398	C5–N3	1.458
C4–C13	1.506	C6–N1	1.322
C2–N2	1.371	N2–C7	1.412
C7–C8	1.400	C8–C9	1.393
C9–C10	1.394	C10–C11	1.394
C11–C12	1.391	C12–C7	1.401
C3–H3	1.081	C6–H6	1.083
C8–H8	1.082	C9–H9	1.084
C10–H10	1.083	C11–H11	1.084
C12–H12	1.085	C13–H13A	1.091
C13–H13B	1.091	C13–H13C	1.091

O1–N3–O2	123.84	O1–N3–C5	117.88	N1–C2–C3	122.17
C2–C3–C4	120.70	C3–C4–C13	118.86	C3–C4–C5	115.92
C4–C5–C6	119.74	C5–C6–N1	123.72	C6–N1–C2	117.73
N1–C2–N2	113.77	C2–N2–C7	129.97	N2–C7–C8	122.17
C7–C8–C9	119.99	C8–C9–C10	120.67	C9–C10–C11	119.35
C10–C11–C12	120.39	C11–C12–C7	120.32	C12–C7–N2	118.50

O1–N3–C5–C6	-15.04	O2–N3–C5–C6	164.36	N1–C2–C3–C4	1.76
C2–C3–C4–C5	-0.50	C2–C3–C4–C13	-179.33	C3–C4–C5–N3	179.09
C4–C5–C6–N1	1.99	C5–C6–N1–C2	-0.77	C6–N1–N2–C7	-179.15
N1–C2–N2–C7	-173.95	C2–N2–C7–C8	41.26	N2–C7–C8–C9	178.14
C7–C8–C9–C10	-1.11	C8–C9–C10–C11	-0.01	C9–C10–C11–C12	0.97
C10–C11–C12–C7	-0.79				

Angle between the plane of NO_2 (O1N3O2)and the plane of pyridine ring(N1,C2-C6)	15.80
Angle between the plane of pyridine ring (N1,C2-C6) and the plane of phenyl ring (C7-C12)	44.70

Table S2. Optimized parameters (Å,°) for 2-*N*-phenylamino-5-nitro-4-methylopyridine HB dimer (PA5N4MP - **2**).

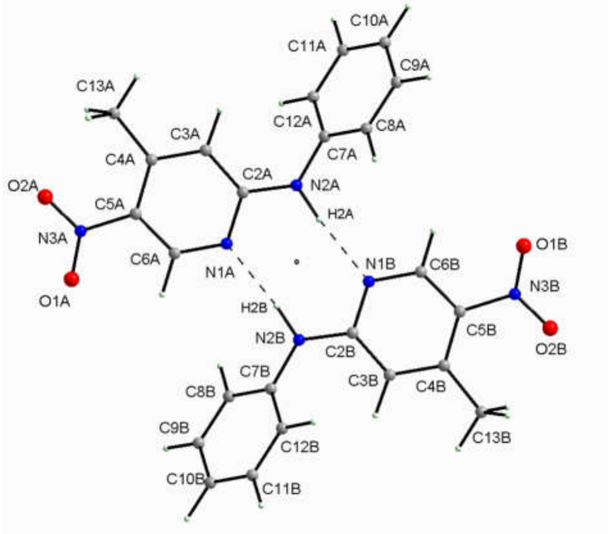

HB dimer $(PA5N3MP)_2$ has an inversion centre

Geometrical parameter for Mol A and Mol B are equal

N3–O1	1.238	N3–O2	1.237
N1–C2	1.360	C2–C3	1.416
C3–C4	1.388	C4–C5	1.421
C5–C6	1.399	C5–N3	1.452
C4–C13	1.507	C6–N1	1.328
C2–N2	1.360	N2–C7	1.419
C7–C8	1.403	C8–C9	1.396
C9–C10	1.397	C10–C11	1.398
C11–C12	1.396	C12–C7	1.403
C3–H3	1.083	C6–H6	1.085
C8–H8	1.086	C9–H9	1.086
C10–H10	1.086	C11–H11	1.086
C12–H12	1.085	C13–H13A	1.093
C13–H13B	1.093	C13–H13C	1.093

O1–N3–O2	123.38	O1–N3–C5	118.10	N1–C2–C3	121.23
C2–C3–C4	121.43	C3–C4–C13	118.73	C3–C4–C5	115.91
C4–C5–C6	119.45	C5–C6–N1	124.08	C6–N1–C2	117.88
N1–C2–N2	115.10	C2–N2–C7	127.45	N2–C7–C8	118.71
C7–C8–C9	120.19	C8–C9–C10	120.30	C9–C10–C11	119.54
C10–C11–C12	120.49	C11–C12–C7	119.98	C12–C7–N2	121.72

O1–N3–C5–C6	0.53	O2–N3–C5–C6	0.98	N1–C2–C3–C4	-0.96
C2–C3–C4–C5	-0.14	C2–C3–C4–C13	179.29	C3–C4–C5–N3	-179.08
C4–C5–C6–N1	-1.39	C5–C6–N1–C2	0.28	C6–N1–C2–N2	179.25
N1–C2–N2–C7	174.02	C2–N2–C7–C8	132.80	N2–C7–C8–C9	176.81
C7–C8–C9–C10	0.85	C8–C9–C10–C11	-0.78	C9–C10–C11–C12	-0.20
C10–C11–C12–C7	1.11				

hydrogen bond in dimer

N2A–H2A	1.029	H2A···N1B	2.005	N2A···N1B	3.030	N2A–H2A···N1B	173.97
N2B–H2B	1.029	H2B···N1A	2.005	N2B···N1A	3.030	N2B–H2B···N1A	173.97

Angle between the plane of NO_2 (O1N3O2)and the plane of pyridine ring(N1,C2-C6) in Mol A 1.20

Angle between the plane of pyridine ring (N1,C2-C6) and the plane of phenyl ring (C7-C12) in Mol A 53.60

Table S3. Optimized parameters (Å,°) for 2-phenylamino-5-nitro-6-methylpyridine (PA5N6MP - **1**).

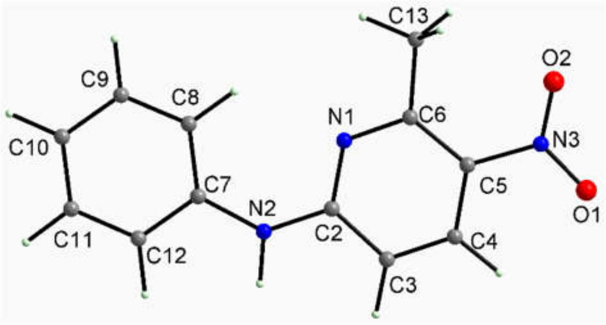

N3–O1	1.230	N3–O2	1.229	N1–C2	1.337
C2–C3	1.417	C3–C4	1.369	C4–C5	1.404
C5–C6	1.408	C6–N1	1.341	C6–C13	1.504
C2–N2	1.372	N2–C7	1.410	C7–C8	1.401
C8–C9	1.394	C9–C10	1.392	C10–C11	1.395
C11–C12	1.383	C12–C7	1.405	N2–H2	1.009

C3–H3	1.084	C4–H4	1.081	C8–H8	1.077
C9–H9	1.084	C10–H10	1.083	C11–H11	1.084
C12–H12	1.086	C13–H13A	1.089	C13–H13B	1.092
C13–H13C	1.092				

O1–N3–O2	123.66	O1–N3–C5	117.60	N1–C2–C3	122.02
C2–C3–C4	118.05	C3–C4–C5	119.59	C4–C5–C6	119.62
C5–C6–N1	119.89	C6–N1–C2	120.84	N1–C2–N2	119.93
C2–N2–C7	132.76	N2–C7–C8	124.68	C7–C8–C9	119.44
C8–C9–C10	121.44	C9–C10–C11	119.03	C10–C11–C12	120.26
C11–C12–C7	120.72	C12–C7–C8	119.13		

O1–N3–C5–C6	180.00	O2–N3–C5–C6	0.00	N1–C2–C3–C4	0.00
C2–C3–C4–C5	0.00	C3–C4–C5–C6	0.00	C4–C5–C6–N1	0.00
C4–C5–C6–C13	180.00	C5–C6–N1–C2	0.00	C6–N1–N2–C7	180.00
N1–C2–N2–C7	0.01	C2–N2–C7–C8	0.00	N2–C7–C8–C9	180.00
C7–C8–C9–C10	0.00	C8–C9–C10–C11	0.00	C9–C10–C11–C12	0.00
C10–C11–C12–C7	0.00				

The conformation of the whole molecule is planar (without H atoms of CH_3).

Table S4. Experimental and calculated wavenumbers with assignment of the observed bands to respective normal modes obtained for the dimeric molecules.

2PA5N4MP			2PA5N6MP			
Calc.	Exp.		Calc.	Exp.		
IR,RS	IR	RS	IR,RS	IR	RS	Assignment
3613vw			3547vs	3344s		νN-H
3536s						νN-H
			3302vs	3224w		νNHO
3237	3240m					νsNHN
3215	3213m					νasNHN
3200vw			3197vw			ν(CH)
3151	3196m					νs(CH)
3142						νs(CH)
3138-3131	3133m		3138vw			ν(CH)
3125-3109			3122vw	3097w	3097vw	νas(CH)
3064		3071w	3087vw			$\nu_s(CH_3)$
3054	3032m		3052vw	3056w	3055vw	$\nu_{as}(CH_3)$
2997	3010m	2995vw	3001vw	2974w		$\nu_s(CH_3)$
	2851m	2840w		2928w	2931vw	$\nu_s(CH_3)$
			1650s	1624m	1646vw	δNHO
1657v		1623m				δasNHN
1648m	1615s	1616w				δsNHN
1610m						δsNHN
			1614m	1609m	1617sh	ν(ϕ)+ν(θ)
1633s	1595vs	1606w	1596m	1598s	1610m	ν(ϕ)+ν(θ)
			1581w	1580s	1586w	ν(NO) + ν(ϕ)
1579m			1577w			ν(ϕ)+ν(θ)
1572m	1571s	1597w				δasNHN
1546m	1562sh					ν(NO) + ν(ϕ)
			1549w	1553m		ν(NO) + δNHO + δCHO
				1543m	1543vw	ν(NO) + δNHO + δCHO
1542m	1543sh	1545w	1536m			ν(NO) + ν(ϕ)
			1503w	1501s	1498w	δCHO
1500m						ν(NO) + ν(ϕ)
			1500w			ν(ϕ)
1499m	1497s	1500w	1490m	1474m	1474w	ν(NO) + ν(ϕ)
			1474w			δCH+δ(CH3)
1473m						δCH
			1471w			δCH+δ(CH3)
1464m		1470w	1452w	1463m	1445w	$\delta(CH_3)$
1463m	1453vs	1446vw				$\delta s(CH_3)$
1451m	1433w	1423vw				ν(ϕ)+$\delta(CH_3)$+δasNHN
			1451w			$\delta(CH_3)$
1450m						$\delta as(CH_3)$
			1449m			$\delta(CH_3)$
1444m						ν(ϕ)+δsNHN
			1442w	1445m		$\delta(CH_3)$
1422w	1419m					ν(C-C)
1396w	1385w	1384vw	1396w	1430w	1398w	$\delta s(CH_3)$
			1364m	1363m	1364w	δ(NHO)+ν(ϕ)+δ(CH3)
1338vs	1338s	1335s				δ(NH)+ν(ϕ)
			1337vs	1358w		δ(CHO)+ν(θ)
1336w						δ(NH)+ν(ϕ)
			1334			δ(CH)
1332vw						δ(ϕ)+δ(θ)
1319vw	1321s	1327vs				$\nu_s(NO_2)$+ν(ϕ)+ $\delta(CH_3)$

		1312s	1309vs	1306vs	1308s	$\nu_s(NO_2)+\nu(\phi)+\delta(CH_3)$
			1296w	1297vs	1300s	$\nu_s(NO_2)+\nu(\phi)+\delta(CH_3)$
1294w	1309m	1293s				$\nu CN+\nu_s(NO_2)$
1283w						δCH
1282w	1279vs	1284s				δCH
				1287vs		$\delta(NCNHO)$
				1277vs	1277vs	$\delta(NCNHO)$
			1251m	1253s		$\delta(NCNHO)$
1253w	1251m	1257m				$\nu(NCN)$
1249w			1245w	1227w	1229w	$\nu(NCN)+\delta NH$
1234w	1237m	1237w				$\nu(C\text{-}NH)$
1231w		1221vw				$\nu(C\text{-}NH)$
			1221w		1207w	$\nu(CN)_{(NH)}$
			1192w	1197w	1197w	$\nu(\phi)$
			1185w	1184w	1182w	δCHO
1183w	1194w	1194w	1183w			δsCH
1176w	1171w	1177w	1172w	1174w	1161vw	δsCH
1161w		1156w				δsCH
1105m	1108m	1108w				$\nu CN_{(NO2)}$
1081w	1078w	1079vw	1130m	1090m		δsCH
			1080vw	1078m	1079w	$\nu CN_{(NO2)}$
1043w	1027w	1028w	1037vw	1031w	1033w	$\delta(CH_3)$
			1032vw			$\delta(CH)+\delta(CH3)$
1027w	1003vw	1004m				$\tau(\theta)$
1024w	960w	962vw				$\delta(CH_3)$
1001w			997vw	996w	997m	δCC
989w			987vw			δCH
			949vw	953w	954w	$\delta(CH)+\delta(CH3)$
916w	912w	912vw	903vw	901w	861m	δCH
873w						$\delta(\phi)+\delta(\theta)$
870w						$\delta(\phi)+\delta(\theta)$
861w	865w	878w				$\delta(CH)+\rho(CH_3)$
	860w					$\delta(CH)+\rho(CH_3)$
834w	837m	836w	854vw	840w	824w	$\delta_s(NO)_\phi+\tau(\phi)$
			829vw			δCH
			821vw	825m	768w	δCH
			815vw			δCH
787w			800w			δ_sNHN
778w						$\delta CNCN$
			773w	767m	756w	$\delta(NO2)+\rho(CH_3)$
767w	763m					δ_sNHN
745w			753w	755m		$\delta(\phi)+\delta(\theta)$
			748w			δCH
743w	743m	776vw	745w			$\delta(\phi)+\delta(\theta)$
736w						δNCN
			728w			δCH
721w						δNCN
	703m	746w				ρNHN
698w		702vw	695w	690m	688m	δCH
			684			$\delta(\phi)+\delta(\theta)$
			674			δNHO
670w	667w					$\tau asNHN$
637w	636w	635vw				$\delta CNCN$
			624w		620m	$\delta(\phi)+\delta(\theta)$
619w	616w	617vw	617		616m	$\delta(\theta)$
			601w			ρCH_3

595w		594vw				$\delta(\phi)+\delta(CH_3)$
561w		564vw	567w		572w	$\delta(\phi)$
		558vw	550			δNH
			535w			$\delta(CH_3)$
529w	528w	530vw	504		508w	$\delta(\phi)+\delta(\theta)$
488	495w	498vw	499			$\delta(\phi)+\delta(\theta)$
487w	460m	460w	445	444w	442w	$\delta(\phi)+\delta(\theta)$
			436w			δCNC
426w						$\delta(\phi)+\delta(\theta)$
412w	430w	415w	410w			$\rho(\phi)+\rho(\theta)$
			392w			$\delta C\text{-}CH_3$
341w		339vw				$\rho(\phi)+\rho(\theta)$
		326vw				$\rho C\text{-}CH_3$
287w			295w			$\rho(\phi)+\rho(\theta)$
			282			δCNC
273w		274vw				$\rho(\phi)+\rho(\theta)$
			258w			δCNC
234w						$\rho(\phi)+\rho(\theta)$
			234w		248w	δCNC
			224w		219w	δCNC
222w		211w				$\tau(CH_3)$
			213w			$\rho(CH_3)$
			211w			$\rho(CH_3)$
202w						$\delta(CNHC)_{\phi+\theta}$
198w						$\delta(CNHC)_{\phi+\theta}$
196w			197w			ρCH_3
190w			196w			ρCH_3
		135sh				$\delta(CNHC)_{\phi+\theta}$
111w						$\delta(\phi)+\delta(\theta)$
104w		102s				$\delta(\phi)+\delta(\theta)$

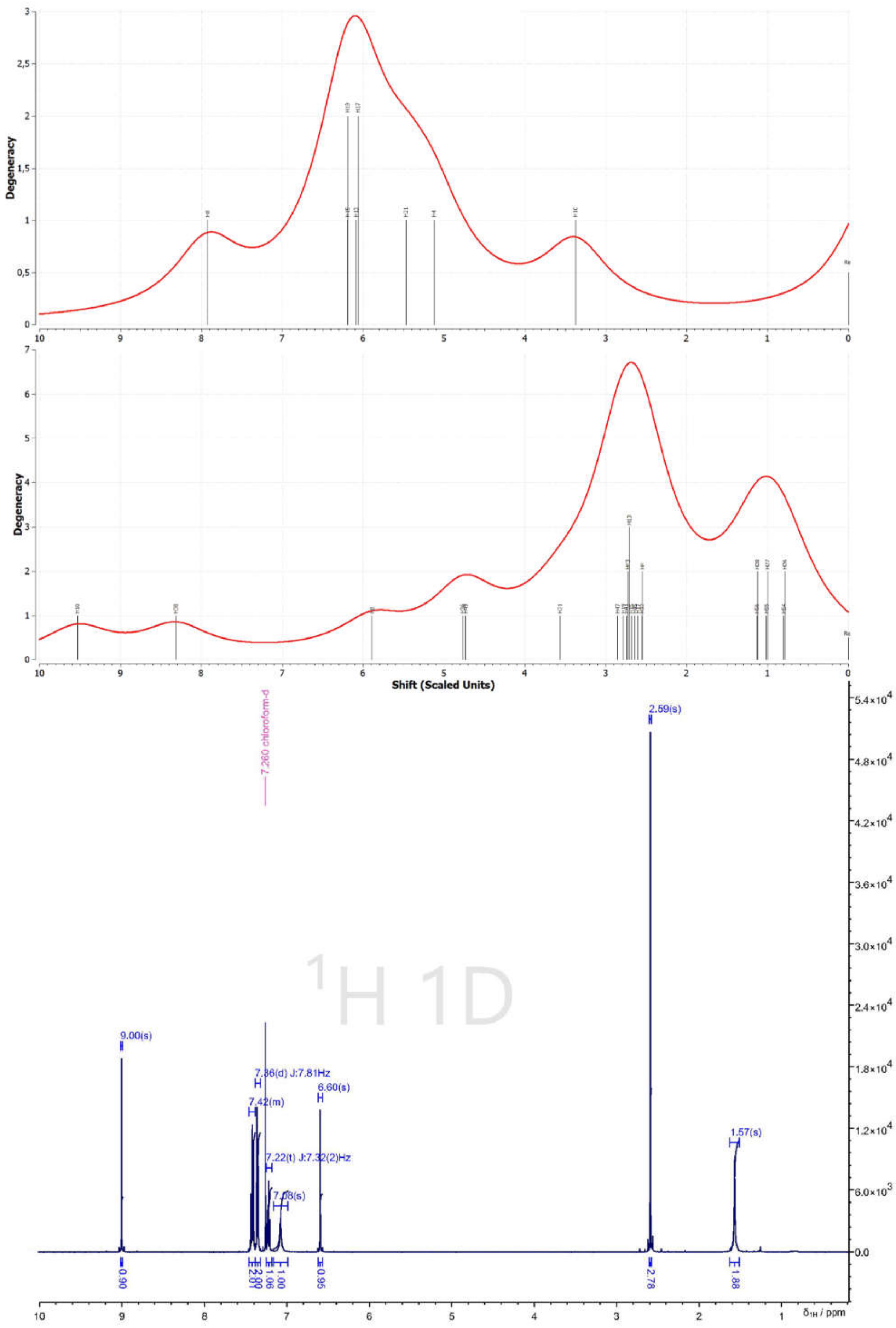

Figure S1. ^{1}H NMR spectra of 2-*N*-pheny lamino-4-methyl-5-nitropyridine.

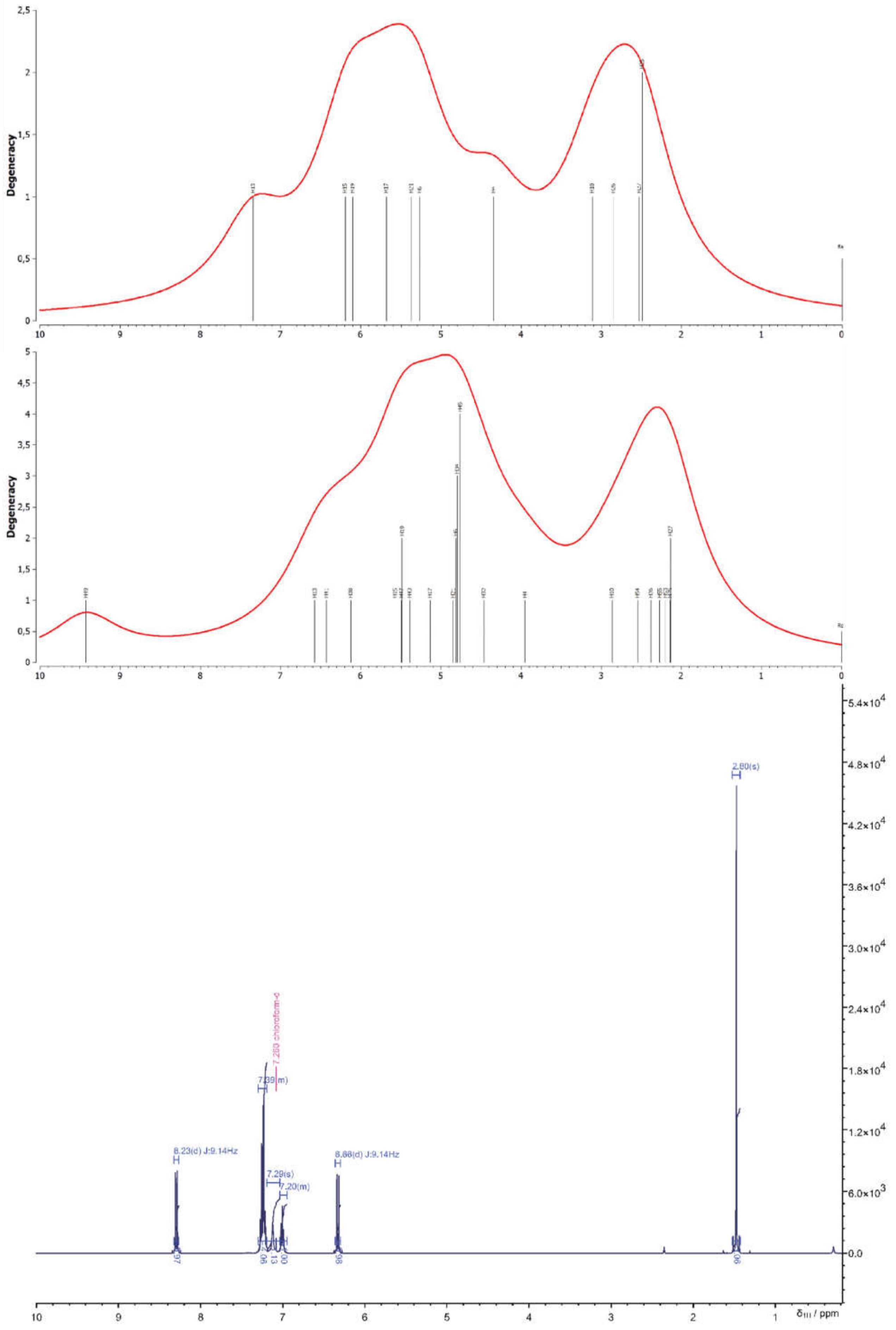

Figure S2. ^{1}H NMR spectra of 2-*N*-phenylamino-6-methyl-5-nitropyridine.

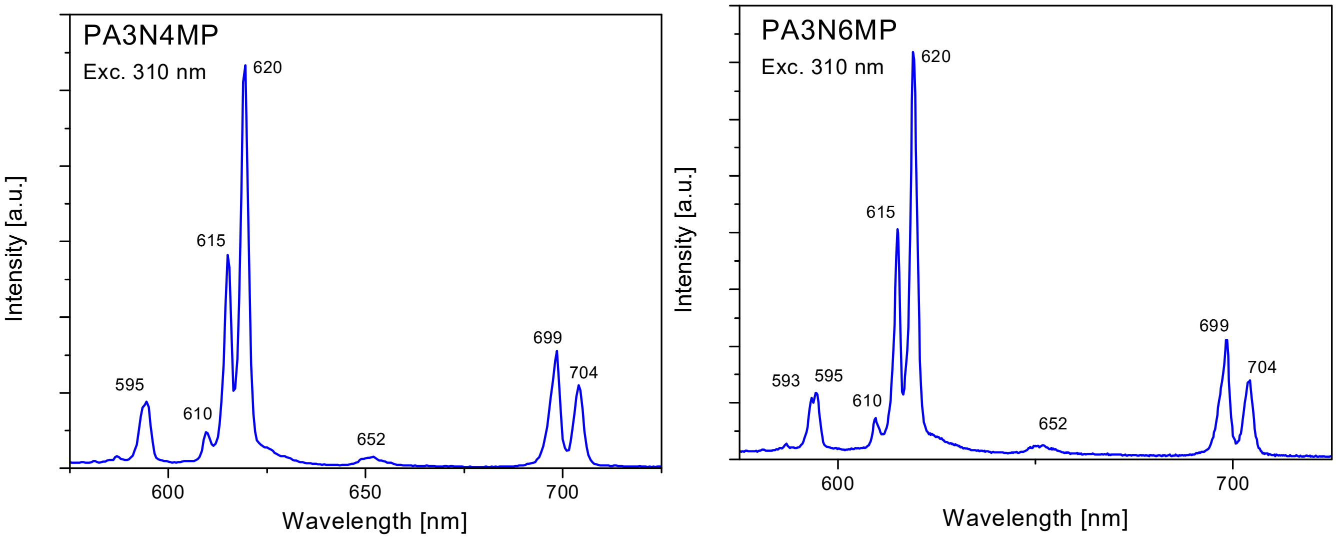

Figure S3. The emission spectra of the europium coplexes obtained in the present work.
